# Mammary Disease during Lactation

**Published:** 1876-03

**Authors:** Salvatore Caro


### MAMMARY DIbEASES DURING LACTATION.

DR. SALVATORE CARO upon "Diseases of the Mammary Gland during Lactation."

After brief reference to enlargement of the gland, and what is known as milk-fever, associated with the establishing of the process of lactation, the author of the paper turned his attention to the consideration of some of the anomalies of this function. Of these agalactia and galactia were first considered in their etiology, symptomatology and treatment. The most reliable remedy for agalactia, according to the author's experience, was the leaves of the plant Ricinis Communis. The powdered beans may be employed in absence of the leaves, but are not nearly as efficacious. In absence of both, *nursing*, good food, etc., are sometimes of great benefit.

With regard to galactia three things should be observed with the view of preventing injury to the mother: (1) Ascertain whether the woman has a well developed nipple or not; (2) whether the infant is healthy and without deformities or not, such as tongue-tie, hare-lip, etc., etc.; (3) whether the woman intends nursing or not.

Haggerty's "Ladies' Companion" was regarded as the best instrument for removing milk from the breast.

Various remedies were mentioned that had been employed for the purpose of arresting the secretion of milk, but the application regarded with the most favor by the author of the paper was ext. bell., gum camph., and stramonium ointment combined. Spread this over the breast every four hours and cover with oil-silk.

Galactocele was next considered. The doctor remarked that he had been in the habit of opening these collections as early as possible, and had been annoyed, as a result of this procedure, by failure in healing of the opening made until the mother had ceased lactation. The aspirator, however, may be resorted to with very great satisfaction. By the use of this instrument nursing need not be interrupted, and danger of rupture of the milk-duct be avoided. The operation can be repeated without inconvenience. Obstruction of lactiferous ducts by colostrum casts was mentioned as a common cause of this difficulty.

Affections of the nipple were next considered somewhat in detail, and the liability of being followed by mammary abscess. Prevention of nipple troubles by attention *previous* to confinement was regarded as being of prime importance. Haggerty's nipple-shield was recommended.

Mastitis is by far the most serious affection, and the general outline of treatment was such as the profession at least *should* be familiar with.

Dr. Garrish spoke somewhat at length upon the subject under discussion. He was of the opinion that ice should *never* be used in cases of mastitis, for the reason that he had seen one case in which *both breasts* were frozen, and subsequently completely sloughed away.

Dr. Lusk favored treatment of galactocele by means of the aspirator. He had never derived very much benefit from the castor-oil leaves in cases of agalactia. He had been much more successful in establishing lactation in such cases by having the breast well stroked from base to nipple by an experienced nurse, and making traction upon the nipple by a breast-pump, than by any method he had ever resorted to. There are cases in which there is lack of development in the acini of the breast, and certainly in such cases applications or treatment of any kind would be useless. In those cases in which it becomes necessary to draw the nipple out, as a general rule, he believed it better to remove the child entirely, than to run the risk of developing abcess by nursing and pulling at the nipple.

When the breast is properly emptied before the third day after confinement, milk-fever is rare. In all those cases in which erosion and fissure of the nipple are present, it is better to remove the child from the breast at once, for there is much less liability of developing abscess if the milk is renewed by some artificial means rather than by permitting the child to nurse.

Dr. Peaslee remarked that there are cases in which it is very desirable to arrest the secretion of milk *entirely*—for example, when the child is still-born.

The important question is, do we know of any method of completely preventing the secretion of milk? He believeed that in any case we may realize an entire arrest of the function of lactation by the use of belladonna.

R.—Aqu. ext. bell., ............................ dr. i.
Aquæ, or
Glycerin ........................................ oz. i.
M.

Keep the breasts constantly wet with this solution, commencing before any signs at all of tumefaction have appeared, is the plan he adopts, and has never had any trouble at all with such cases.

Dr. Sell remarked that occasionally he had found it difficult to arrest the secretion of milk by the use of belladonna alone. In such cases he had had admirable success, (1) by enjoining abstinence from fluids as much as possible; (2) by giving a few doses of iodide of potassium, in addition to the belladonna. By these three things combined he had *never* failed to produce perfect agalactia.

With regard to mammary abscess, prophylaxis was regarded as the essential element. Sore nipples being a frequent cause, they must be carefully looked after, and not only that, but one step farther should be taken, and guard against the occurrence of sore nipples. He mentioned the fact that he had seen eight thousand successive cases of confinement in one hospital in Europe, and not a single mammary abscess among them all, for the reason that prophylactic treatment was rigidly observed.

Dr. Hubbard remarked that in case the child was still-born, he did nothing at all with the breast, except to apply camphorated oil and lead-plaster perhaps, and let them entirely alone. He had never had any trouble with this plan of management.

Dr. Lusk sustained the remarks of Dr. Hubbard.

The Academy then adjourned.

